# Lactic control of tumor growth: a new role for an old metabolite

**DOI:** 10.1038/s41392-023-01565-7

**Published:** 2023-08-16

**Authors:** Carolina E. Hagberg

**Affiliations:** 1 Division of Cardiovascular Medicine, Department of Medicine Solna, Karolinska Institutet, Stockholm, Sweden; 2 Center for Molecular Medicine, Karolinska Institutet, Stockholm, Sweden

**Keywords:** Cancer metabolism, Cell biology

In a recent study published in *Nature*, Liu and co-workers show that high lactate production by tumor cells promotes premature mitotic escape and further cell proliferation by facilitating the binding of Ubiquitin Conjugating Enzyme E2 C (UBE2C) to the anaphase promoting complex (APC/C).^1^ This is important as it suggests cancer metabolism not only to be a consequence of tumor growth, but also one of its drivers.

**Fig. 1:**
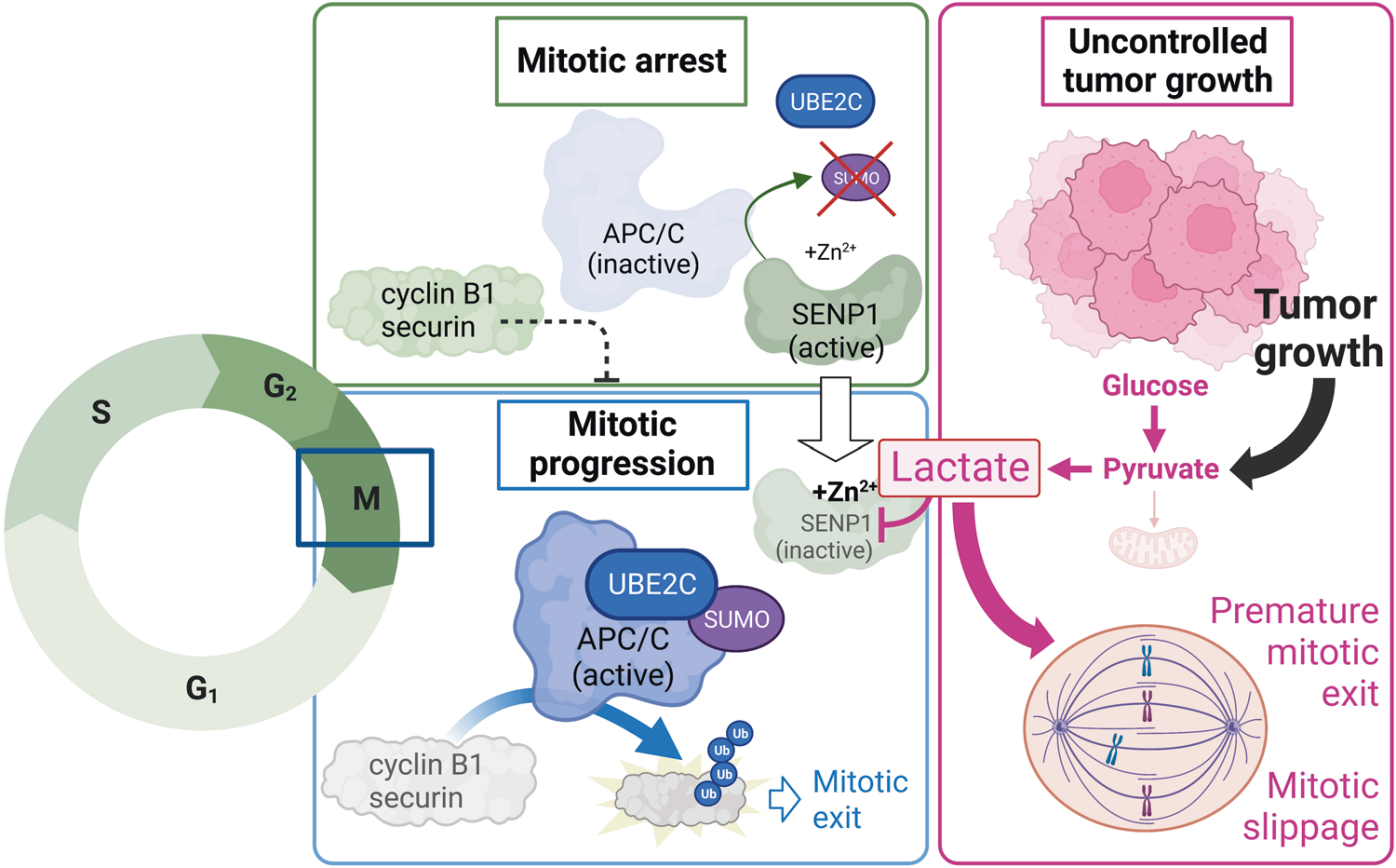
Lactate connects tumor metabolism to cell cycle progression. During mitotic arrest, APC/C is maintained in its inactive form via continuous deSUMOylation by SENP1, which prevents UBE2C binding to APC/C and its subsequent activation (green box). Liu et al. demonstrate that lactate produced by tumor cells can inactivate SENP1 by enhancing the inhibitory effect of zinc ions binding to the SENP1 active site. This inhibition stabilizes SUMOylation of the APC/C subunit APC4 (not shown), which facilitates UBE2C binding to APC/C, APC/C activation and subsequent ubiquitination of its substrates including cyclin B1 and securing (blue box). Importantly, the authors show that increased lactate production thereby is able to drive premature mitotic exit and mitotic slippage, contributing to uncontrolled tumor growth and progression (pink box). Created with BioRender.com

Since its initial discovery over 200 years ago, lactate has sustained the interest of a wide range of research fields due to its significance as energy fuel, redox buffer and signaling molecule.^2^ High quantities of lactate are produced through glycolysis in muscle during exercise, through bacterial lactic acid fermentation, and perhaps most famously, by rapidly growing tumor cells. However, lactate is also the metabolic end-product and/or energy substrate for a range of human cell types under physiological conditions, as exemplified by lactate shuttling between neurons and astrocytes, and many other cell types.^3^ Importantly, lactate has been shown to play several non-metabolic roles, inducing for example histone lactylation, and thereby altering the transcription of a range of genes.^4^ Lactate can also signal by binding to the G-coupled receptor HCAR1 (formerly known as GPR81), which inhibits the lipolytic activity of adipocytes and thereby reduces circulating fatty acid levels during intense exercise.^2^ As shown in the recent article by the lab of Ed Chouchani, published in *Nature* on March 10, 2023, lactate can now add *cell cycle regulation* to its impressive list of biological functions^1^ (Fig. 1).

Treating tumor cell lines with the metabolically active enantiomer L-lactate for 15 min to identify previously unrecognized functions for the metabolite, the team found one of the most affected proteins to be UBE2C, a cell cycle-associated ubiquitin-conjugating enzyme. UBE2C stood out as it is the binding partner of APC/C, a key regulator of cell cycle progression and metaphase-to-anaphase transition. Binding of UBE2C to APC/C leads to the ubiquitination and destruction of a range of cell cycle effector proteins such as cyclin B1 and securin, allowing cells to exit the mitotic checkpoint and proceed towards cell division. The team led by first author Weihai Liu show in a series of elegant biochemical and biophysical experiments that high levels (15 mM) of sodium L-lactate, but not equimolar amounts of its enantiomer D-Lactate or of sodium chloride, can promote premature mitotic checkpoint escape by enhancing the binding of UBE2C to APC/C. Specifically, they unveil an intricate cascade of events that is initiated by lactate forming an inhibitory complex with zinc ions in the active site of SENP1, a protease that helps retain cells at the mitotic checkpoint by continuously deSUMOylating APC/C. The binding of zinc and lactate to SENP1 inhibits its activity, which stabilizes APC/C SUMOylation levels and leads to APC/C undergoing the necessary conformational changes to allow UBE2C binding and the subsequent ubiquitin-mediated degradation of cyclin B1 and securin. The authors go on to show that across a range of cancer cell lines, a higher mitotic rate is associated with increased intracellular lactate levels, and that endogenous lactate production is enhanced during the later phases of cell cycle progression, although it would have been nice to see the levels in unsynchronized cultures as a control. Reducing endogenous lactate levels by either inhibiting lactate formation, or increasing lactate by blocking its export, greatly affected mitotic progression, with increased levels of lactate promoting premature cell cycle progression and enhanced cellular proliferation. Most importantly, the authors show that lactate accumulation could allow tumor cells to prematurely escape even a mitotic blockage induced by chemotherapy agents targeting microtubules, by allowing cyclin B1 to be degraded and cell cycle to proceed despite the absence of correct microtubule assembly, a well-known phenomenon termed mitotic slippage. Although the bulk of the presented data stems from cultured cancer cell lines, the findings may, if confirmed in vivo and in patient samples, open up a renewed interest for using so-called aerobic glycolysis inhibitors to lower tumor lactate production in parallel with more cytotoxic chemotherapy treatments.

In addition to comprehensively delineating the somewhat complex mechanism of lactate-mediated cell cycle regulation in great detail, a strength of the current study is the correct use of controls for osmolarity throughout the study. By using both equimolar amounts of D-lactate, pyruvate or sodium chloride as controls, the study avoids some of the recently highlighted pitfalls of accidentally altering cellular molarity when supplying living systems with large quantities of sodium L-lactate.^5^ However, while the present study offers great biochemical detail into the binding of lactate to the SENP1 active site and subsequent cellular consequences, it does leave some questions open. For one, as the study uses only cultured cancer cell lines, and shows little results from primary cells, it remains unknown if the revealed mechanisms are only active in transformed cancer cells in culture, or also are translatable to tumors in vivo, and/or to primary non-transformed cells that produce high levels of lactate. Examples of the latter include not only hypertrophic adipocytes mentioned by the authors, but also neurons, activated immune cells and stem cells. Does lactate produced during cell growth function as a universal signal for a beneficial cellular environment for cell division, or is it merely another metabolite hijacked by tumor cells to serve their ultimate purpose? While the altered and highly glycolytic metabolism of rapidly growing cells to date have been considered mainly a consequence of rapid cellular proliferation, the data presented by Liu and colleagues now suggests the converse to be possible too, with growth-activated lactate production by itself promoting cell cycle progression. More research is clearly needed to understand the exact cellular and environmental conditions that allow lactate to highjack mitotic control, and why such a regulatory function would exist in normal cells. Despite lactate being our first and perhaps also best-recognized cancer-associated metabolite, its potential new effects on mitotic cell cycle progression might, upon validation, produce a much-needed renaissance in the interest to develop drugs that target lactate synthesis and uptake, giving new attention to our oldest star within cancer metabolism.
